# Ultra-stable CsPbBr_3_ Perovskite Nanosheets for X-Ray Imaging Screen

**DOI:** 10.1007/s40820-019-0283-z

**Published:** 2019-06-24

**Authors:** Liangling Wang, Kaifang Fu, Ruijia Sun, Huqiang Lian, Xun Hu, Yuhai Zhang

**Affiliations:** 1 Collaborative Innovation Center of Technology and Equipment for Biological Diagnosis and Therapy in Universities of Shandong, Institute for Advanced Interdisciplinary Research (iAIR), University of Jinan, Jinan, 250022 People’s Republic of China; 2 School of Physics and Technology, University of Jinan, Jinan, 250022 People’s Republic of China; 3 School of Material Science and Engineering, University of Jinan, Jinan, 250022 People’s Republic of China

**Keywords:** CsPbBr_3_, Perovskite, Nanosheets, Self-assembly, X-ray imaging screen

## Abstract

Wet chemistry methods, including hot-injection and precipitation methods, have emerged as major synthetic routes for high-quality perovskite nanocrystals in backlit display and scintillation applications. However, low chemical yield hinders their upscale production for practical use. Meanwhile, the labile nature of halide-based perovskite poses a major challenge for long-term storage of perovskite nanocrystals. Herein, we report a green synthesis at room temperature for gram-scale production of CsPbBr_3_ nanosheets with minimum use of solvent, saving over 95% of the solvent for the unity mass nanocrystal production. The perovskite colloid exhibits record stability upon long-term storage for up to 8 months, preserving a photoluminescence quantum yield of 63% in solid state. Importantly, the colloidal nanosheets show self-assembly behavior upon slow solidification, generating a crack-free thin film in a large area. The uniform film was then demonstrated as an efficient scintillation screen for X-ray imaging. Our findings bring a scalable tool for synthesis of high-quality perovskite nanocrystals, which may inspire the industrial optoelectronic application of large-area perovskite film.

**Electronic supplementary material:**

The online version of this article (10.1007/s40820-019-0283-z) contains supplementary material, which is available to authorized users.

## Highlights

- A gram-scale CsPbBr_3_ perovskite nanosheet colloid was synthesized by a green method at room temperature.
- The perovskite nanosheet colloid shows uncompromised photoluminescence quantum yield upon storage for over 8 months.
- A self-assembly crack-free thin film of the colloidal nanosheets demonstrated an efficient X-ray imaging screen.

**Electronic supplementary material:**

The online version of this article (10.1007/s40820-019-0283-z) contains supplementary material, which is available to authorized users.

## Graphical abstract

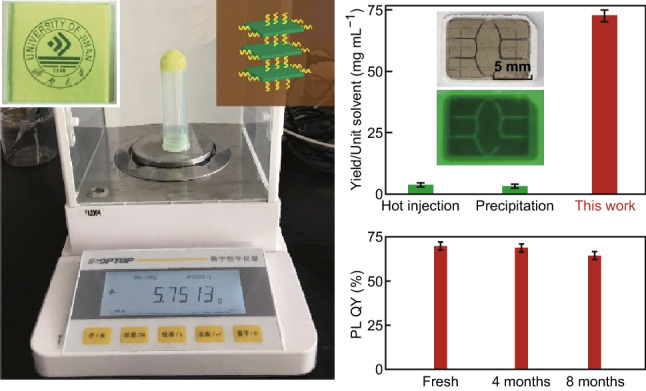

## Introduction

Thin-film optoelectronic devices based on lead halide perovskite materials have demonstrated remarkable performance due to their exceptional optical and electronic properties [1–5]. For example, MAPbI_3_-based solar cells have achieved a record efficiency of 24.2% according to the US national renewable energy laboratory [6, 7], and CsPbBr_3_-based light-emitting diodes (LED) have also reached a high efficiency exceeding 20% by using MABr as an additive [8]. As a potential scintillator, all-inorganic perovskite nanocrystals (NCs) have recently displayed many significant advantages over conventional scintillating materials. For example, Chen et al. [9] reported a solution-processed scintillator based on cesium lead halide perovskite NCs, which show both a low detection limit and a multicolor tuning ability.

A scalable synthetic method for high-quality colloidal perovskite is the rule of thumb for practical use of perovskite-based optoelectronic devices [10]. Since the first report of all-inorganic perovskite NCs in 2015, a variety of methods have been devised for their synthesis or even scalable production, including hot-injection [11, 12], room temperature re-precipitation [13, 14], emulsion methods [15], and template-assisted routes [16]. These wet chemistry-based methods usually require a large amount of solvent for dispersion, but generate a very small quantity of NC solids [10]. For example, to obtain 3 mg of a CsPbBr_3_ NC solid, the hot-injection method requires 1000 mg of a high boiling point solvent, which is eventually disposed of as environmental waste [11, 12]. Additionally, in such methods, solely upscaling the precursor is not feasible because it would compromise on the product quality. Thus, such a synthesis is neither cost-effective nor environment-friendly. Many methods have been proposed to upscale the synthesis with minimum solvent expense. For example, mechanically induced crystallization, such as ball milling [17] and ultrasonic-assisted synthesis [18], has been attempted to achieve low-cost gram-scale production of perovskite NCs. Yet, the resulting NCs show limited stability upon long-term storage [17].

Halide-based perovskite NCs show intrinsic vulnerability to moisture and irradiation with light, suppressing their active performance in optoelectronic devices [19]. To increase their stability, a common strategy is to use functional ligands to graft the surface of NCs. Galian and Pérez-Prieto et al. [20] reported MAPbBr_3_ perovskite NCs with an extended storage stability by using 2-adamantylammonium bromide (ADBr) as the only capping ligand. Alternatively, core–shell construction is proven to be an effective passivation method. Chen et al. [21] reported a heterodimer of CsPbX_3_/ZnS which shows a 12-fold enhancement in storage stability in air; Meyns et al. used poly(maleic anhydride-*alt*-1-octadecene) (PMA) as a passive shell and found that 70% emission intensity was maintained over a 12-h irradiation period, while the untreated NCs kept only 20% of their original intensity [22]. Despite this progress, long-term storage (> 6 months) stability of perovskite colloids has not yet been reported.

Herein, we report an environment-friendly synthesis of a CsPbBr_3_ nanosheet colloid with both high reaction yield and long-term storage stability. The synthesis affords a solid yield of 70 mg NCs out of 1-mL solvent, which is over 20-fold higher than the conventional hot-injection method. The as-synthesized colloid can be kept in a capped vial for over 8 months while retaining 94% of its original photoluminescence quantum yield (PL QY) (~ 68%) as a result of both the quantum size effect and PbBr_2_ passivation. Importantly, both the crystal phase and the emission band could be fine-tuned by changing the feeding ratio of Pb/Cs. The self-assembly behavior of nanosheets not only allows a careful investigation of the energy transfer process between thin and thick nanosheets, but also leads to the formation of a crack-free thin film where an X-ray imager can be demonstrated.

## Experimental

**Fig. 1:**
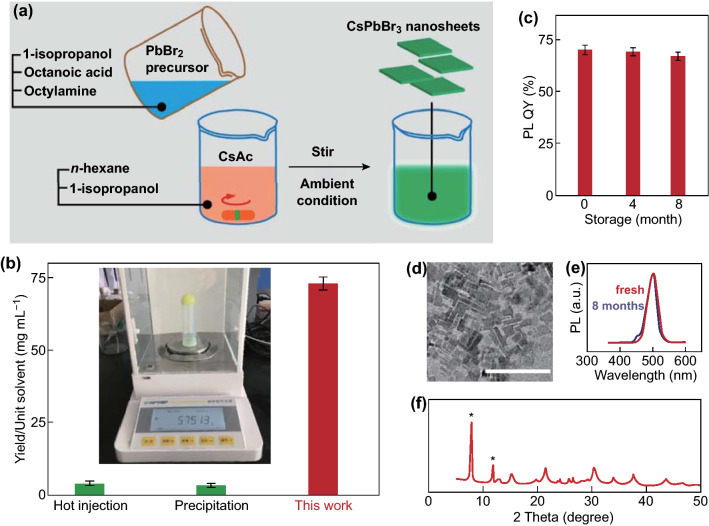
**a** Schematic showing the synthetic procedure of ultra-stable CsPbBr_3_ NCs. **b** Reaction yield comparison between this experiment and conventional methods, such as hot-injection and precipitation. The inset shows 5.75 g of as-synthesized nanosheets generated in a 100-mL beaker. **c** PL QY measurements of both fresh and aged (stored in a capped vial) colloidal samples, indicating a robust storage stability. The error bar was obtained by three parallel measurements. **d** TEM image of the nanosheets. Scale bar represents 100 nm. **e** PL spectra of fresh and aged samples, showing no significant difference. **f** XRD pattern of self-assembled nanosheets, showing a cubic phase. The peaks marked with asterisks indicate the inter-plane spacing of assembled nanosheets

**Fig. 2:**
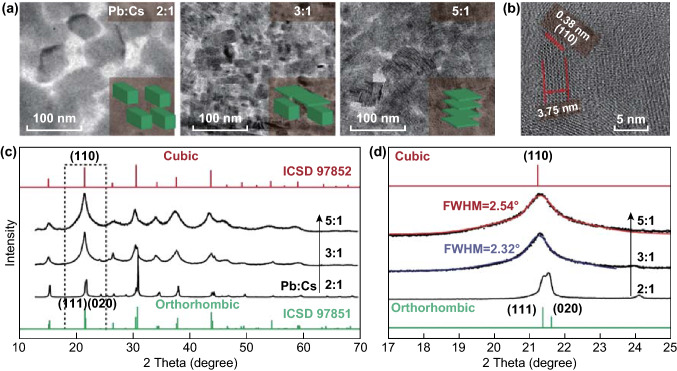
Phase engineering of CsPbBr_3_ NCs through feeding varied molar ratios of Pb/Cs. **a** TEM images showing morphology transformation from bulk cubes to thin nanosheets. **b** High-resolution TEM image of nanosheets (Pb/Cs = 5/1). **c** XRD patterns showing a phase evolution from orthorhombic to cubic. **d** The magnified XRD peaks show a clear difference in peak width, indicating a shrinkage of grain size

The CsPbBr_3_ NCs were synthesized via a modified precipitation method (Fig. 1a) [23, 24]. First, PbBr_2_ was dissolved in a mixture of 1-isopropanol, octanoic acid, and octylamine, generating a PbBr_2_ precursor. Second, a cesium precursor was prepared by vigorously stirring a combination of cesium acetate, *n*-hexane, and 1-isopropanol. The reaction was then initiated by loading the PbBr_2_ precursor into the cesium precursor at room temperature under ambient conditions (see the supporting information for further details). A bright green color appears immediately, indicating the formation of CsPbBr_3_ NCs. The NC has an irregular platelet shape with a diagonal size of 21 nm (Figs. 1d and S1) and a thickness of 3.75 nm (Figs. 2b and S5).

## Results and Discussion

Conventionally, high-quality CsPbBr_3_ NCs require multiple-step purification to ensure the complete removal of both the chunky by-product and excessive solvent [25], eventually leading to a large loss of reaction yield. For example, either the hot-injection method or precipitation synthesis affords less than 3 mg solid out of a 1-mL solvent input [12], which generates a large quantity of organic waste, serving as an environmental pollutant. Meanwhile, a polar solvent is usually used to break the colloidal stability, leading to a severe deterioration of perovskite NCs. Remarkably, through low-speed centrifugation (< 4000 rpm) without the involvement of any polar solvent, our synthesis is able to produce up to 70 mg out of a 1-mL solvent, which is over 20-fold higher than conventional methods, such as hot-injection and precipitation (Fig. 1b). In addition, such polar-solvent-free purification imparts much enhanced stability upon colloidal storage. The colloidal sample was kept in a capped vial for 8 months and 94% of the PL QY remained from its fresh counterpart (Fig. 1c), which is a record stability, to the best of our knowledge. It is worth noting that 99% of the original PL QY (68%) was preserved during the first 4 months of storage.

To understand how the perovskite NCs were stabilized, both the size and phase were engineered by adjusting the feeding ratio of Pb/Cs. It has been proven that the particle size is closely related to the phase stability in perovskite system [26]. For example, Swarnkar et al. [26] observed that the NC form of CsPbI_3_ at room temperature can be preserved as a cubic-phase perovskite, which is only stable in bulk form at high temperatures, and they reasoned that the large surface energy of NCs may provide additional stability. Our findings are consistent with such observations (Fig. S2). As shown in Fig. 2a, the particle gradually changes from 70-nm polygons to 21-nm nanosheets (Figs. 2b and S1) with an increasing feeding ratio. The reduced size was also reflected from the broadening of XRD peaks, where the average crystalline dimension can be calculated according to the Scherrer equation. As shown in Fig. 2d, the full width at half maximum increased from 2.32° to 2.54°, indicating a thickness decreasing from 3.4 to 3.1 nm. These values are in good agreement with the HRTEM result (Fig. 2b).

**Fig. 3:**
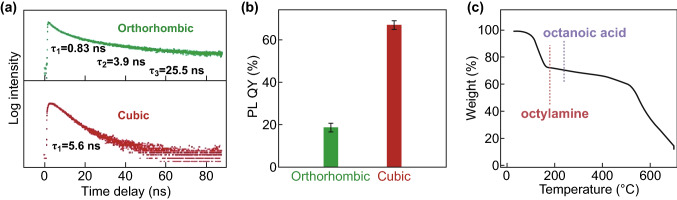
**a** Lifetime measurement at 520 nm and **b** PL QY of CsPbBr_3_ NCs, showing a phase dependence. A 410-nm pulsed laser was used as excitation source, and the PL bands from 450 to 600 nm were monitored as emission. **c** TGA of nanosheets shows ample surface coverage of ligands

Along with the decrease in size, the crystallite phase simultaneously changed from orthorhombic to cubic, as evidenced by the peak merging at 21.4° and 21.6° (Fig. 2c, d). This can be ascribed to the re-arrangement of the crystal lattice from a slightly tilted octahedral network to an ordered array, whereby optical properties largely vary. As shown in Fig. 3a, the photoluminescence lifetime of orthorhombic NCs comprises three components, including 0.83, 3.9, and 25.5 ns. The corresponding sample shows a PL QY of 18.5% (Fig. 3b), indicating the existence of abundant traps or defect states where nonradiative transition occurs. In stark contrast, the cubic-phase NCs show a mono-exponential decay curve, featuring a lifetime of 5.6 ns. Such nanosheets exhibit a high PL QY up to 68%, suggesting that most excitons recombined through a radiative process where quantum confinement dominates in thin nanosheets. The TGA measurement shows that about 25% of the weight was contributed to the ligands, equivalent to ~ 8 nm^−2^ in ligand density (see supporting information for details). Judging from the boiling point, the nature of the ligand is more likely to be octylamine than octanoic acid. The ample coverage of the ligands, together with the amorphous PbBr_2_ passivation (Fig. S7), imparts the high PL QY of nanosheets.

**Fig. 4:**
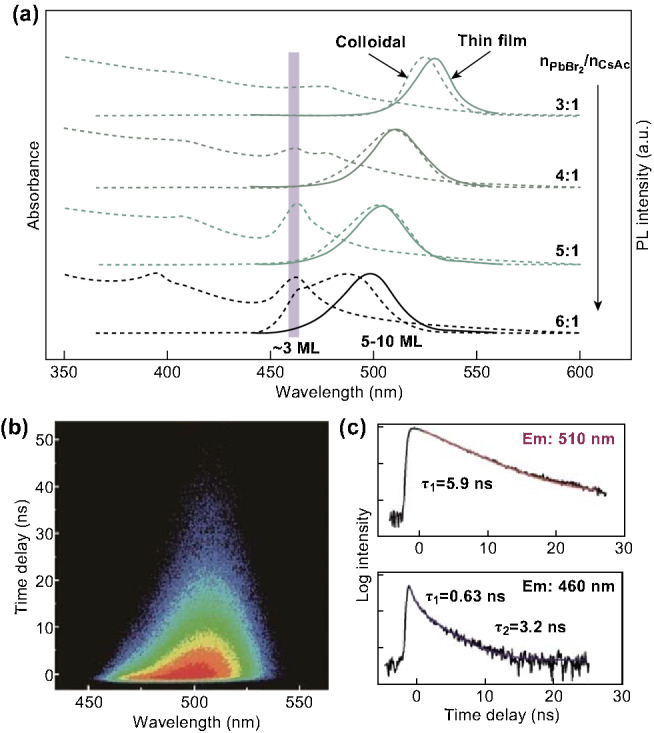
**a** Absorption and photoluminescence spectra of CsPbBr_3_ NCs, showing the PL fine-tuning ability of our synthesis. The purple stripe highlights the excitonic peak of the 3-monolayer nanosheets. **b** 2D pseudo-color transient emission map from CsPbBr_3_ nanosheets colloid (Pb/Cs = 5/1). Streak camera measurement was taken under excitation at 410 nm, flux at 1 pJ cm^−2^. **c** PL decay traces extracted at 460-nm and 510-nm window (± 5 nm)

A current challenge in perovskite-based LEDs is the dearth of pure green emitting thin film, dubbed as the “green gap” [27–29]. Although a polycrystalline thin-film displays a pure green emission at 530 nm, the PL QY of these bulk perovskites is usually very low (< 0.5%) [30]. While the NC solids of all-inorganic perovskites far outperform their polycrystalline counterparts, they have a blueshift emission at 510 nm. Encouragingly, our synthetic method provides a flexible tool to fine-tune the emission bands, fully covering the green gap. As shown in Fig. 4a, the emission bands of colloids gradually shift from 500 to 530 nm as the feeding ratio decreases. It is worth noting that the absorption profile shows multi-exciton features, indicating the admixture nature of samples comprising different numbers of PbBr_6_^−^ monolayers (ML). The absorption peak at 460 nm is ascribable to the 3-ML CsPbBr_3_ nanosheet, which is in good agreement with the previous report [31]. The 5–10-ML nanosheets give rise to the absorption continuum from 470 to 530 nm as a result of weakened quantum confinement. Note that PL QY is slightly compromised over emission tuning yet retains a value over 40% (Fig. S3).

**Fig. 5:**
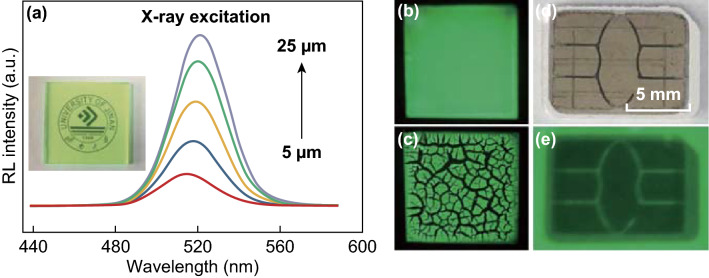
X-ray scintillation screen based on CsPbBr_3_ nanosheets. **a** Radioluminescence spectra of thin films with varied thickness. The inset shows a semitransparent thin-film sample of a flat surface. Radioluminescent image of **b** a crack-free screen and **c** a cracked screen. **d** Photograph and **e** X-ray image of a SIM card

Intriguingly, the nanosheet samples show a much larger Stokes shift (~ 55 nm) than their nanocube counterparts (< 5 nm) [25]. Such a red shift has recently been observed in perovskite NC assemblies, where an inter-NC electronic coupling effect was pronounced [32]. Indeed, the nanosheets in this study had a strong tendency to stack closely into an ordered assembly due to their topological features. To test the self-assembly behavior, the concentrated colloid was cast into a thin film (Fig. 5a), and the small-angle XRD pattern was collected. The sharp peaks at 3.9°, 7.9°, and 11.8° evidenced an assembly of layered structures with an inter-plane distance of 2.2 nm (Fig. S4).

The nanosheets show a red shift in PL after solidification as shown in Fig. 4a. This can be explained by the close proximity between nanosheets with different numbers of MLs, where efficient energy transfer is encouraged (Fig. S6). In fact, such energy transfer processes can even be observed in colloids, where re-absorption dominates over electronic coupling [33].

To quantitatively evaluate the energy transfer process in the nanosheet colloid, a streak camera measurement was taken. As shown in Fig. 4b, the PL intensity distribution is a distorted Gaussian profile, indicating the heterogeneous nature of the samples. The PL bands at 510 and 460 nm were extracted, and the corresponding lifetimes were fitted, as shown in Fig. 4c. The 510-nm band exhibits a mono-exponential decay with a typical lifetime of 5.9 ns, which is in good agreement with the overall lifetime of the 450–600 nm regime. In stark contrast, the lifetime of the 460-nm band shows bi-exponential decay with both a short component of 0.63 ns and a long component of 3.2 ns. The long component is consistent with Akkermann’s report where 3-ML CsPbBr_3_ nanoplatelets show a monomolecular decay with a 3-ns lifetime [31]. It is reasonable to infer that the donor–acceptor energy transfer process gives rise to the short component. Such a bimolecular model can be viewed as a Förster resonance energy transfer (FRET) system, where the FRET efficiency (*E*) is calculated to be about 80% by Eq. 1 [34]:

1 $$E = 1 - \frac{{\tau_{\text{da}} }}{{\tau_{\text{d}} }}$$

where *τ*_da_ and *τ*_d_ are the lifetimes of the donor–acceptor conjugate and the pristine donor, respectively. As can been seen, the FRET between nanosheets is extremely efficient. This could be explained by the nature of the assembly in the colloidal state, which can be evidenced by the fact that even low-speed centrifugation (~ 4000 rpm) is sufficient to precipitate the nanosheets.

The efficient energy transfer provides an energy cascade avenue to produce efficient radioluminescence (RL) [9]. Indeed, the thin film of CsPbBr_3_ nanosheets not only exhibits a bright photoluminescence under UV excitation, but also shows a slight red-shifted radioluminescence under X-ray exposure (Fig. 5a, b). Simultaneously, a red shift of radioluminescence was observed from 515 to 520 nm, with the film thickness increasing from 5 to 25 μm, which is ascribed to enhanced re-absorption. The strong green emission region corresponds well with the most sensitive region of commercial charge-coupled device (CCD), which inspired us to construct a simple X-ray imager. As a proof-of-concept experiment, the nanosheets were drop-cast onto a thin glass slide and allowed to dry naturally at room temperature to form a flat film. Note that drying at an elevated temperature (50 °C) leads to the generation of large cracks (Fig. 5c). The flat film was then used as a projection screen to register the spatial information carried by the transmitted X-ray [24]. In a typical trial, a subscriber identification module (SIM) card was imaged by our prototype X-ray imager, and the internal structure can be seen clearly, with a relatively high resolution of ~ 330 μm (Figs. 5d, e and S8).

## Conclusions

We presented a green synthesis for gram-scale production of CsPbBr_3_ nanosheets colloid. The colloid shows uncompromised PL QY upon storage for over 8 months due to the size effect and PbBr_2_ passivation. Importantly, the synthesis allows one to fine-tune the emission bands of perovskite NCs, readily covering the “green gap.” The preferred cubic phase can be simultaneously facile controlled at room temperature. Through streak camera measurement, we eventually identified an efficient energy transfer process between thin and thick nanosheets, leading to the exhibition of bright radioluminescence. The X-ray imager was showcased with a relatively high resolution. Our findings hold promise for the commercialization of high-performance perovskite NCs, which may be beneficial for the application in the light converting or X-ray imaging industries.

## Electronic supplementary material

Below is the link to the electronic supplementary material.

Supplementary material 1 (PDF 886 kb)
