# Supplementary material for: Ultra-stable CsPbBr3 Perovskite Nanosheets for X-Ray Imaging Screen
Source: Nanomicro Lett. 2019 Jun 24;11:52. doi: 10.1007/s40820-019-0283-z (PMC7770729; doi:10.1007/s40820-019-0283-z)
Supplement: Supplementary file 1 — Supplementary material 1 (PDF 886 kb) [file 40820_2019_283_MOESM1_ESM.pdf]

Supporting Information for

Ultra-stable $CsPbBr_3$ Perovskite Nanosheets for X-ray Imaging Screen

Liangling Wang[1, 2], Kaifang Fu[1], Ruijia Sun[1], Huqiang Lian[3], Xun Hu[3], Yuhai Zhang[1,*]

[1]Collaborative Innovation Center of Technology and Equipment for Biological Diagnosis and Therapy in Universities of Shandong, Institute for Advanced Interdisciplinary Research (iAIR), University of Jinan, Jinan 250022, People's Republic of China

[2]School of Physics and Technology, University of Jinan, Jinan 250022, People's Republic of China

[3]School of Material Science and Engineering, University of Jinan, Jinan 250022, China

*Corresponding author. E-mail: ifc_zhangyh@ujn.edu.cn (Yuhai Zhang)

S1 Experimental Methods

S1.1 Chemicals

Lead (II) bromide ($PbBr_2$, 99.0 %) and caesium acetate (CsAc, 99.99% trace metals basis) were purchased from Aladdin. Octylamine (OcAm, 99%) and octanoic acid (OcAc, 99%) were purchased from Macklin. 1-propanol (PrOH), n-Hexane (Hex, 99.5%), and toluene (TOL, 99.5%) were purchased from Tianjin Fuyu Fine Chemical Co., Ltd, China. All chemicals were used without any further purification.

S1.2 Fabrication and Purification of $CsPbBr_3$ Nanocrystals (NCs)

The fabrication of $CsPbBr_3$ NCs was conducted by using a modified method reported by G. Konstantatos et al. [S1]. Specifically, 384 mg of CsAc,12 mL of 1-PrOH and 24 mL of Hex were loaded into a 100 mL vial and stirred in air at ambient temperature firstly. Then, 3.67 g $PbBr_2$ was added into a 20 mL vial with 5.4 mL of 1-PrOH, 5.4 mL OcAc and 5.4 mL OcAm and heated at 90 °C in air under vigorous stirring. After complete dissolution of the $PbBr_2$ salt, the hot solution was quickly injected into the prepared CsAc solution under vigorous stirring at ambient condition. The color of the mixture turned greenish immediately, and 2 min later the reaction completed. Finally, the mixture was centrifuged at 4,000 rpm, and the wet pellet of the NCs was dispersed in 36 mL toluene for further characterization.

S1.3 Photoluminescence and Absorption

The as-prepared nanocrystals were diluted for 100 times in n-hexane for steady-state measurements of photoluminescence and absorption, respectively. A Cary5000 UV-Vis spectrometer (Agilent Technologies) was used for absorption measurements in the range from 450 to 700 nm. A FluoroMax-4 spectrofluorometer (Horiba Scientific; a slit width of 2 nm and a scan rate of 500 nm min^{-1}) was used to record the photoluminescence spectra. The excitation wavelength used for the$CsPbBr_3$ nanocrystals were set at 400 nm for thin film and 365 nm for colloid, respectively. The PLQY data were collected on in an integrated sphere coupling on a steady state PL spectrometer (Edinburg FS5), under an excitation at 365 nm. Three trials on thin-film samples were conducted and results were averaged to present the final PL QY values.

S1.4 Transmission Electron Microscopic Measurement

Transmission electron microscopic (TEM) images were acquired using a Tecnai transmission electron microscope with an acceleration voltage of 120 keV. HRTEM images were acquired using the same instrument. Low-dose HRTEM images were acquired by a Gatan K2 direct-detection camera operated in electron-counting mode (camera counting frame rate of 400 fps (frames per second) at 4 k × 4 k resolution) with a final image output rate of 40 fps at 4 k × 4 k resolution. An exposure of 3 s therefore results in an image stack of 120 individual image frames. These frames can be summed to improve the signal-to-noise ratio (SNR).

S1.5 X-ray Diffraction Measurements

Powder X-ray diffraction (XRD) was performed using a Bruker AXS D8 diffractometer with Cu-Ka radiation (λ = 1.5406 Å). The samples were prepared via the drop casting of the nanocrystal suspension onto a clean glass slide, followed by drying at room temperature.

S1.6 Streak Camera Measurement

Time-resolved photoluminescence color map was collected using a high-resolution streak camera (Hamamatsu C10910) where the pump beam is generated with the second harmonic (410 nm) of a Spectra-Physics MaiTai eHP and Inspire HF-100 OPO. APE Pulse Select pulse picker is used to select the repetition rate of the pulse beam to 4 MHz. Measurements were performed at room temperature with the excitation fluence of 1 pJ cm^{-2}.

S1.7 Radioluminescence and X-ray Imaging

Radioluminescence were obtained by an Edinburgh FS5 fluorescence

spectrophotometer (Edinburgh Instruments Ltd., UK) equipped with a miniature X-ray source (AMPEK, Inc.). The photographs of X-ray-excited luminescence were acquired with a digital camera (Canon 600D with EF-S 35mm f/2.8 IS STM lens).

S2 Ligands Density Calculation

In order to figure out the ligands density on the $CsPbBr_3$ nanosheets, the TGA measurement was performed (Fig. 3c). The ligand is found to contribute about 25% to the total weight. The nature of ligand is found to be octylamine according to the boiling points of octylamine.

If we assume that a $CsPbBr_3$ nanosheet with a size of 21×21×3 nm^3 (Pb:Cs = 5:1), the mass of a nanosheet can be obtained by Eq. S1

$$m_{CsPbBr_3} = \rho abc = 6.28 \times 10^{-18} g \quad \text{(S1)}$$

where ρ is the density of $CsPbBr_3$ (4.75g cm^{-3}), *a*, *b* and *c* are the length of a $CsPbBr_3$ nanosheet along three directions, respectively.

The TGA result expresses a mass relation between a $CsPbBr_3$ nanosheet and the ligands on its surface. Hence, we get the mass of ligands on a $CsPbBr_3$ nanosheet as shown in Eq. S2:

$$m_{ligands} = \frac{25\%}{75\%} m_{CsPbBr_3} = 2.09 \times 10^{-18} g \quad \text{(S2)}$$

Then, the total number of ligands $N_{ligands}$ on a $CsPbBr_3$ nanosheet can be obtained according Eq. S3:

$$N_{ligands} = \frac{m_{ligands}}{M_{ligands}} N_A = 9.74 \times 10^3 \quad \text{(S3)}$$

where $M_{ligands}$ is the molar mass of octylamine, N_A is the Avogadro constant.

Thus, the density of ligands on the $CsPbBr_3$ nanosheet can be got according Eq. S4

$$D_{ligands} = \frac{N_{ligands}}{2(ab+bc+ac)} \approx 8\ /nm^2 \quad \text{(S4)}$$

S3 Supplementary Figures

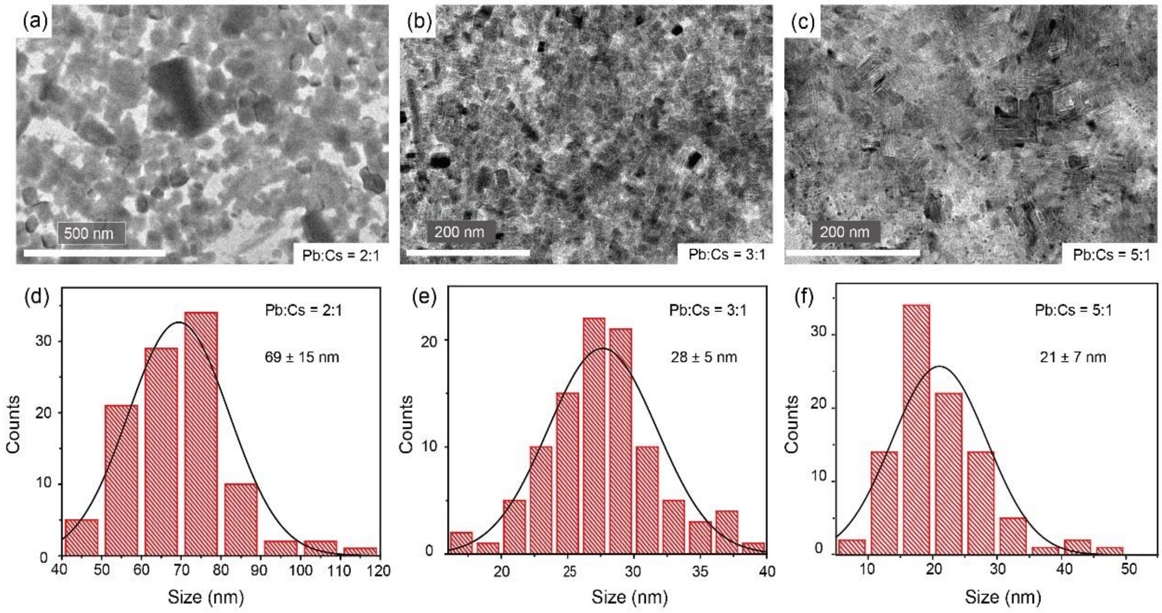

Fig. S1 TEM image of $CsPbBr_3$ NCs with Pb:Cs = 2:1 (**a**), 3:1 (**b**) and 5:1 (**c**). Size distribution of $CsPbBr_3$ nanosheets with different Pb:Cs ratio of 2:1 (**d**), 3:1 (**e**) and 5:1 (**f**). Note that diagonal length instead of thickness was counted for 100 particles from a typical TEM image.

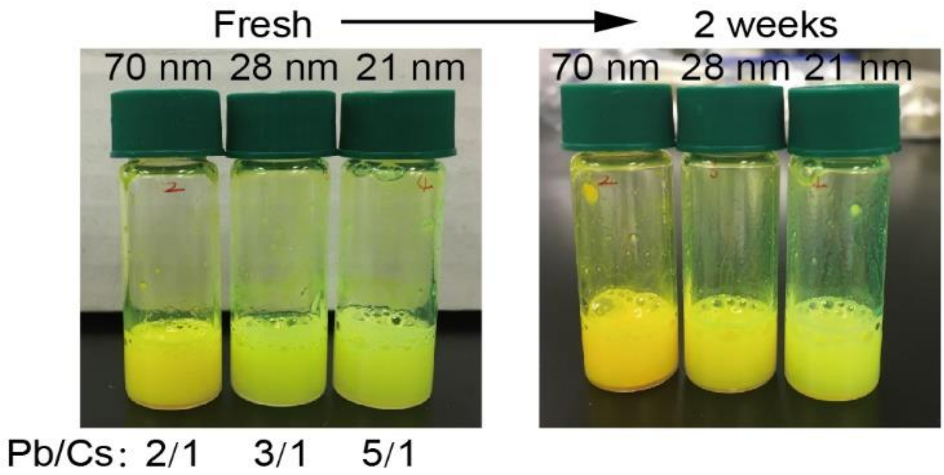

Fig. S2 Photographs showing $CsPbBr_3$ NC colloids of varied stability which is induced by size difference. Both 70-nm and 28-nm samples turned yellow, which is less active in PL. In contrast, 3-nm-thick nanosheets (21 nm in diagonal length) remains green.

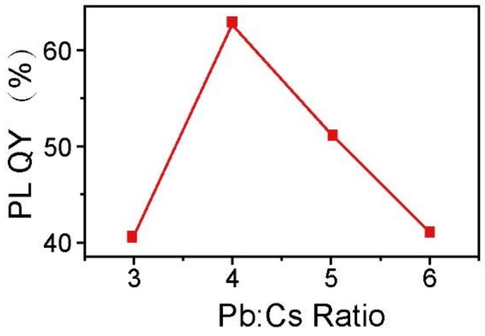

Fig. S3 PLQY measurement of $CsPbBr_3$ NC colloids synthesized at varied feeding ratio of Pb/Cs

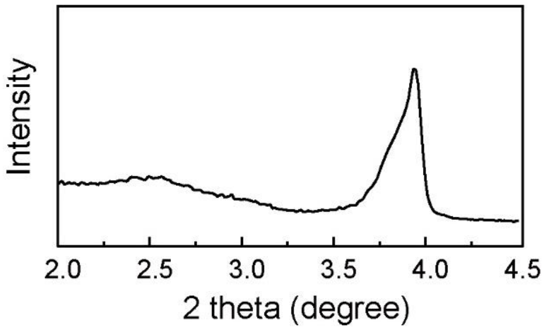

Fig. S4 Small angle XRD of $CsPbBr_3$ NC sample synthesized at Pb/Csfeeding ratio of 4/1, showing an inter-plane diffraction peak at 3.9°

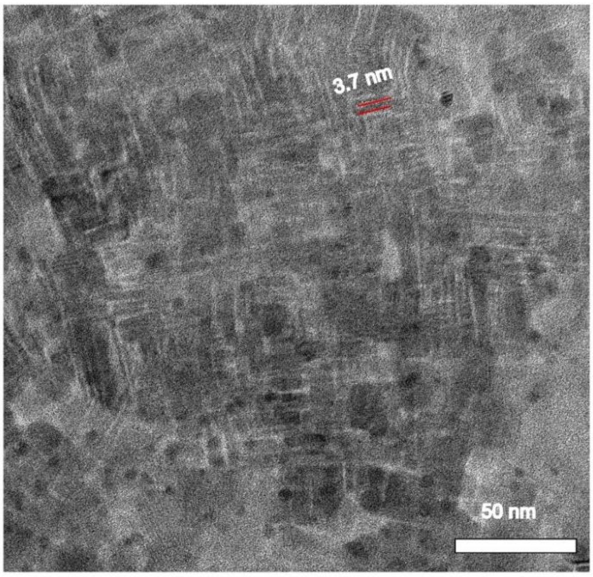

Fig. S5 TEM image of $CsPbBr_3$ NCs (Pb:Cs = 5:1) showing a pattern of assembly of nanosheets, the thickness of a nanosheet is about 3.7 nm.

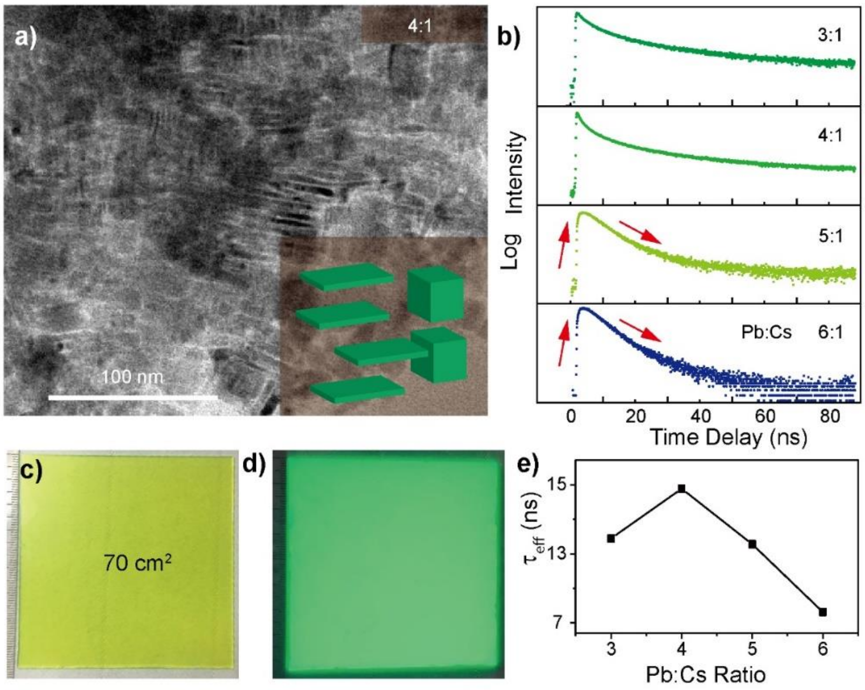

Fig. S6 a TEM image of $CsPbBr_3$ NCs showing a less ordered pattern of assembly of nanosheets and polygons. **b** PL lifetime traces of $CsPbBr_3$ NCs synthesized at different ratio of Pb/Cs. The extracted lifetime was plotted in **e**. The building-up stages at 5:1 and 6:1 samples indicate an efficient energy transfer process due to more ordered pattern of assembly. **c** Photograph of large-area thin film on glass slide, featuring a flat and crack-free surface and **d** the corresponding luminescent image under 365-nm lamp illumination

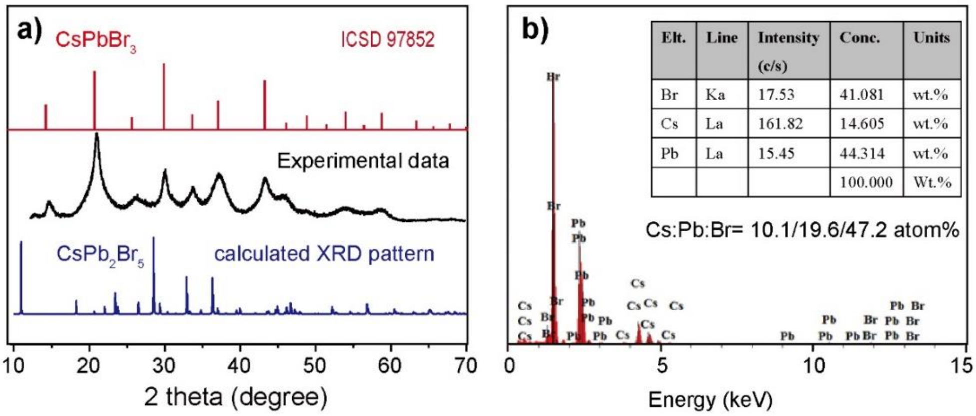

Fig. S7 a XRD pattern comparison between $CsPbBr_3$ and $CsPb_2Br_5$, and the experimental data clearly matches with $CsPbBr_3$ crystalline. **b** the energy dispersive spectrum of sample, showing an elemental composition of $CsPb_2Br_5$. However, based on the XRD patterns in **a**, the excessive $PbBr_2$ are likely amorphous and acts as a passivation layer to $CsPbBr_3$ nanosheets.

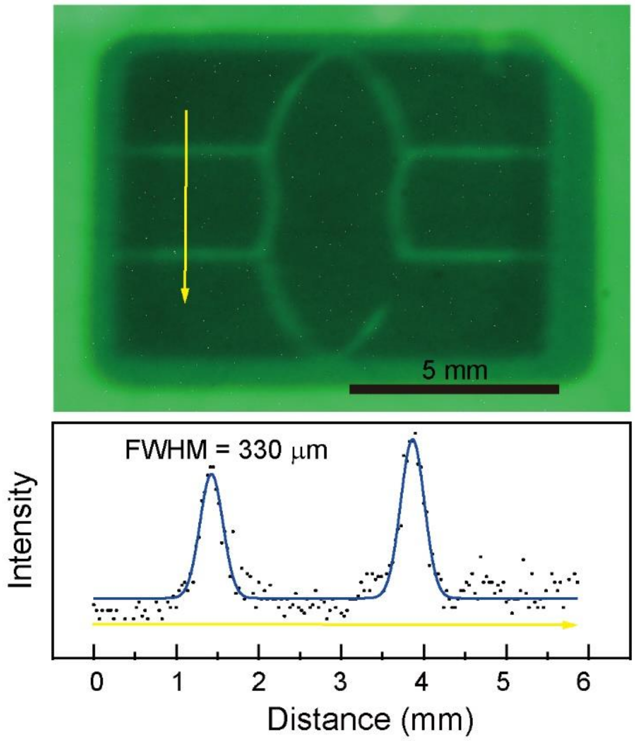

Fig. S8 Spatial resolution measurement by fitting of intensity spread profile with Gaussian function. The FWHM was taken as resolution. The yellow arrow show the data trace of collection.

Supplementary Reference

[S1]F. Di Stasio, S. Christodoulou, N. Huo, G. Konstantatos, Near-unity photoluminescence quantum yield in $CsPbBr_3$ nanocrystal solid-state films via postsynthesis treatment with lead bromide. Chem. Mater. **29**(18), 7663-7667 (2017). https://doi.org/10.1021/acs.chemmater.7b02834
